# Semantic and Syntactic Processing During Comprehension: ERP Evidence From Chinese QING Structure

**DOI:** 10.3389/fnhum.2021.701923

**Published:** 2021-12-24

**Authors:** Siqin Yang, Yeyi Cai, Wen Xie, Minghu Jiang

**Affiliations:** ^1^Center for Psychology and Cognitive Science, Tsinghua University, Beijing, China; ^2^Department of Politics and International Relations, University of Oxford, Oxford, United Kingdom

**Keywords:** Chinese, sentence, semantic, syntactic, ERPs, N400, LAN, P600

## Abstract

Previous studies used BA and BEI structures as stimuli to infer that syntax-first models seemed not applicable in Chinese. However, there were inconsistent results of both within same structures and between different structures. Since sentence structures of stimuli were non-canonical as well as lacking wide representativeness in Chinese, we examined the processing mechanism of a more representative structure in Chinese, QING (QING + NP_1_ + V + NP_2_) structure in the current study. Four conditions, including correct sentences (CORRECT), semantic-violated sentences (SEMANTIC), syntactic-violated sentences (SYNTACTIC), and combined violated sentences (COMBINED), were composed by manipulating the V between NP_1_ and NP_2_. Results with respect to three types of violation were as follows. In the initial phrase (100–300 ms), there existed an interaction between SEMANTIC consistency and the SYNTACTIC category. In the intermediate phrase (300–500 ms), the interaction continued with similar negative waves evoked by three types of violated sentences. In the final phrase (500–700 ms), both SYNTACTIC or COMBINED evoked obvious negative waves. The current research of Qing structure provided new evidence for the processing mechanism of Chinese sentence patterns. Specifically, we found that the interactive model rather than the syntax-first model may apply to the processing of this specific structure of Chinese sentences and compared the results with those reported in previous studies that examined other types of sentence structures.

## Introduction

Comprehension of sentences of individuals is closely correlated with semantic analysis and syntactic anatomy. A neurocognitive model assumes that sentence comprehension might be divided into three phases, phase 1 (100–300 ms), phase 2 (300–500 ms), and phase 3 (500–1,000 ms) (Friederici, 2002), with syntactic processing rather than semantic processing dominant during sentence comprehension (the syntax-first model). Previous research used Chinese Subject–Ba–Object–VP (S + Ba + O + V) structure (Ba structure) (Ye et al., 2006) to find that Chinese sentence comprehension could also be subdivided into different phases and contradicted the syntax-first model with Object–Bei–Subject–VP (O + Bei + S + V) structure (Bei structure) (Yang et al., 2015; Zeng et al., 2020). Since results of these structures appeared inconsistent, and more frequent canonical structures in Chinese, (S + V + O) structure (Zhang et al., 2013) has been rarely examined, the aim of the current study was to examine the processing mechanism of the typical (S + V + O) structure, Qing–Subject–VP–Object structure (Qing structure) and compare the results with those reported in previous studies that examined other types of sentence structures.

The syntax-first model (Frazier and Fodor, 1978; Friederici, 2002) assumes that local syntactic structures based on word category information are built independent of lexical–semantic information but not *vice versa* during the first stage. During the second stage, thematic role assignments proceed. If the initial syntax and the theme do not match, reanalysis takes place in the third stage. In contrast, the interactive model (Marslen-Wilson, 1975) claims that syntactic processing and semantic processing already interact at an early stage. Friederici (2002) proposed a new model with three phases in the light of electrophysiological data and neuro-topographical specifications on brain-imaging evidence. During phase 1 (100–300 ms), the initial syntactic structure is formed on the basis of the word category, which is similar to the syntax-first model. During phase 2 (300–500 ms), lexical-semantic and morphosyntactic processing takes place together for the goal of thematic role assignment, which is partial to the interactive model. During Phase 3 (500–1,000 ms), different types of information are integrated.

Whether syntactic processing is dominant in the initial phase has been controversial across different languages. Since event-related potentials (ERPs) contain three particular ERP components, N400 component, left anterior negativity (LAN), and P600 component closely related to sentence comprehension, they are regarded as ideal technologies to address this issue in different languages. N400 component is a negative deflection peaking around the 400 ms post-stimulus onset, with a centro-parietal distribution (Yang et al., 2021). It appeared in the processing of incongruent words in both sentential and single-word contexts (Kutas and Hillyard, 1980; Mayerhofer and Schacht, 2015). Because N400 was exclusively sensitive to semantic contents that become part of episodic memory traces, it has been considered to reflect lexical integration processing, instead of automatic lexical priming (Brown and Hagoort, 1993; Friederici et al., 1999). In addition, the N400 component was believed to be a valid marker for semantic integration processing (Friederici et al., 1999; Friederici, 2002; Ye et al., 2006; Zhang et al., 2013; Yang et al., 2015; Zeng et al., 2020).

Left anterior negativity appeared in a very early time window. When it occurred during the time window of 100–200 ms, it was also named as Early LAN (ELAN). This component has been found to reflect phrase structure and word category violations (Friederici et al., 1993, 1996; Hagoort et al., 2003; Vincenzi et al., 2003). Moreover, the LAN with peak latency during 300 and 500 ms has been observed in response to morphosyntactic violations (Friederici et al., 1993, 1996) in English (Coulson et al., 1998), German (Friederici et al., 1999), Hebrew (Deutsch and Bentin, 2001), Italian (Vincenzi et al., 2003), and Spanish (Martin-Loeches et al., 2006). P600 component is a positive deflection starting at about the 500 ms post-stimulus onset and lasting several 100 ms, with centroparietal and, sometimes, frontal distributions. It was initially found in reading a “garden-path sentence” (temporarily ambiguous or puzzling as it contains a word group that appeared to be compatible with more than one structural analysis) (Osterhout and Holcomb, 1993). It was also found following the ELAN or LAN in syntactically violated sentences, such as phrase structural and morphosyntactic violations (Friederici et al., 1996; Coulson et al., 1998; Hagoort et al., 2003; Vincenzi et al., 2003). Hence, its amplitude was claimed to be a valid marker for syntactic integration difficulty (Kaan et al., 2000) and syntactic reanalysis (Friederici, 1995; Delogu et al., 2019). Numerous studies used P600 amplitude to detect whether there is syntactic processing or not (Friederici et al., 1999; Friederici, 2002; Ye et al., 2006; Zhang et al., 2013; Yang et al., 2015; Zeng et al., 2020).

What is more, P600 effect was also reported to be associated with semantic processing and reanalysis, which was also called “semantic P600” (Chow and Phillips, 2013; Yang et al., 2015). Such positive amplitude has two reported origins seemingly different from the normal P600 effect. First, it arises in a grammatically correct yet role-reversed sentence, which was interpreted as the implausibility of the sentence meaning (e.g., 高材生把数学题难倒了。 Translation: The student baffled the math problem) (Chow and Phillips, 2013). Second, sentence structures with either a verb-noun mismatch (e.g., 小赵修理一张信纸。 Translation: Zhao repaired a piece of writing paper.) or a double mismatch (e.g., 小赵修理一台信纸。 Translation: Zhao repaired a set of writing paper.) also stimulated semantically related P600 effect. In this case, the positive amplitude was interpreted as the initiation of semantic processing coordination at diversely hierarchical syntactic levels (Jiang and Zhou, 2012). Especially, if the semantic process at the basic level encounters intricate obstacles, the cognitive processing system may generate a new process, transferring from this basic semantic-processing level to another more advanced level, in order to comprehend this context. These three ERPs components were widely recognized as typical components to reflect the processing mechanism of sentence comprehension.

Friederici et al. (1999) supported the syntax-first model by using German as language materials to explore this issue. The researcher made four sets of sentence conditions (a). CORRECT, Die/Wand/wurde/bunt/bemalt (Translation: The wall was colorfully painted over); (b). SEMANTIC, Die/Suppe/wurde/manchmal/bemalt (Translation: The soup was sometimes painted over); (c). SYNTACTIC, Die/Wand/wurde/vom/bemalt (Translation: The wall was by the painted over); (d). COMBINED, Die/Suppe/wurde/zur/bemalt (Translation: The soup was for the painted over). Results revealed that a significant N400 effect appeared in SEMANTIC, while withheld in SYNTACTIC and COMBINED. The absence of N400 effect for SYNTACTIC was interpreted as syntactic violation not interfere with semantic processing. As there was also no N400 effect observed in COMBINED, these results seemed to indicate that semantic processing reflected by the N400 was affected by initial phrase structure building (syntax) associated with the early anterior negativity (similar to LAN). Based on such evidence, it claimed that the syntax-first model was authentic during sentence comprehension in German. Similar results also have been found in other Indo-European language studies (Ferreira and Clifton, 1986; Gunter et al., 1997, 2000; Ainsworth-Darnell and Shulman, 1998; Friederici, 2002, 2011; Hahne and Friederici, 2002).

However, previous Chinese studies provided counter-evidence to the syntax-first model in the initial phase. Ye et al. (2006) conducted four sets of sentence conditions by using Chinese Ba structure, (a). CORRECT, 设计师制作新衣, 把布料裁了。 Chinese Pinyin: She/ji/shi/zhi/zuo/xin/yi, ba/bu/liao/cai/le. English translation: To make new dresses, the stylist cut the cloth. (b). SEMANTIC, 伐木工开采森林, 把松树裁了。 Chinese Pinyin: Fa/mu/gong/kai/cai/sen/lin, ba/song/shu/cai/le. English translation: Exploiting/the/forest, the/timber/jack/cut/pine/trees. (c). SYNTACTIC, 设计师制作新衣, 把裁了。 Chinese Pinyin: She/ji/shi/zhi/zuo/xin/yi, ba/cai/le. English translation: To make new dresses, the stylist cut. (d). COMBINED, 伐木工开采森林, 把裁了。 Chinese Pinyin: Fa/mu/gong/kai/cai/sen/lin, ba/cai/le. English translation: Exploiting the forest, the timberjack cut. SYNTACTIC elicited an early anterior negativity (similar to LAN), merging into a sustained anterior negativity, and then a broadly distributed negativity appeared during P600 times window instead of positive component. SEMANTIC evoked an early starting N400. COMBINED revealed an early anterior negativity similar to that of SYNTACTIC, however with a larger negativity. The absence of P600 in both SYNTACTIC and COMBINED may be caused by a component overlap between the posterior positivity and the broadly distributed negativity (Ye et al., 2006). The authors inferred that, in the initial phase, the syntactic processing and semantic processing were parallel and independent in contrast to the syntax-first model. Then, an interaction of syntax and semantics appeared during the intermediate phase, which seemed to support the interactive model.

In addition to BA structure, BEI structure also provided solid pieces of evidence against the syntax-first model in the initial phase (Zhu et al., 2018). Yang et al. (2015) conducted BEI structure with similar four conditions (a). CORRECT, 那块玻璃被蒋娜仔细地擦拭了多遍。 Chinese Pinyin: Na/kuai/bo/li/bei/jiang/na/zi/xi/de/ca/shi/le/duo/bian. English translation: That piece of glass is carefully wiped by Na Jiang many times.). (b). SEMANTIC, 那个方案被胡杰仔细地擦拭了多遍。 Chinese Pinyin: Na/ge/fang/an/bei/hu/jie/zi/xi/de/ca/shi/le/duo/bian. English translation: That plan is carefully wiped by Jie Hu many times. (c). SYNTACTIC, 那块玻璃被蒋娜仔细地抹布了多遍。 Chinese Pinyin: Na/kuai/bo/li/bei/jiang/na/zi/xi/de/ma/bu/le/duo/bian. English translation: That piece of glass is carefully dishcloth by Na Jiang many times. (d). COMBINED, 那个方案被胡杰仔细地抹布了多遍。 Chinese Pinyin: Na/ge/fang/an/bei/hu/jie/zi/xi/de/ma/bu/le/duo/bian. English translation: That plan is carefully dishcloth by Jie Hu many times. Results showed that both N400 and P600 appeared in three violated sentences, unlike the case in Indo-European languages where N400 is absent in COMBINED. These results conveyed that the syntax-first model was not always feasible in Chinese sentence comprehension.

Nevertheless, there were two limitations in existent Chinese experimental studies, (i) inconsistent results of both similar and different structures and (ii) sentence structure of stimuli being non-canonical and lacking widely representativeness in Chinese. Both aspects were reviewed below. First, Ba (S + Ba + O + V) structure and Bei (O + Bei + S + V) structure were used as stimuli in previous studies. In similar BA structures, Ye et al. (2006) observed N400 in SEMANTIC, LAN, and negative waves instead of P600 in both SYNTACTIC and COMBINED. Ye et al. (2007) observed N400 in SEMANTIC and larger N400 in SYNTACTIC, yet no LAN and P600. Yu and Zhang (2008) observed both N400 and P600 in SEMANTIC and COMBINED without LAN. Zhang et al. (2010) observed different N400 components in three violated sentences and P600 in COMBINED, yet LAN was absent in three violated sentences. In similar BEI structures, one study reflected that SYNTACTIC and COMBINED evoked the consistent P600 effect (Yang et al., 2015), while another study showed that the amplitude of SYNTACTIC was more positive than that of COMBINED (Zeng et al., 2020). What is more, there was an obvious LAN in BA structures (Ye et al., 2006). However, LAN was not significant (or not reported) in BEI structures (Yang et al., 2015; Zeng et al., 2020). These incongruent results in a similar structure may be caused by their different words. Since the Ba (S + Ba + O + V) and Bei (O + Bei + S + V) structure contain a prominent syntactic marker Ba and Bei in the middle of their sentences with totally different word orders, these different pieces of evidence in different structures might be also caused by difference in either word orders or syntactic markers. In a word, only BA and BEI structures may not be representative enough to claim that the syntactic processing and semantic processing were parallel and independent, or the syntax-first model definitely did not exist in Chinese.

Secondly, for Indo-European language sentences, the syntax-first model has been verified in widely used sentence structures and in different languages in the same family (Ferreira and Clifton, 1986; Gunter et al., 1997, 2000; Ainsworth-Darnell and Shulman, 1998; Friederici, 2002, 2011; Hahne and Friederici, 2002). In contrast, these stimuli of previous Chinese research, including Ba (S + Ba + O +V) structure, Bei (O + Bei + S + V) structure, and object–subject–verb structure (O + S + V structure) (Zhang et al., 2013), are non-canonical and not representative in Chinese. Since the results of these structures were inconsistent, and one of the most frequent canonical structures in Chinese, (S + V + O) structure (Zhang et al., 2013) has been rarely studied; the present study chose the typical (S + V + O) structure, QING (QING + NP_1_ + V + NP_2_) structure as experimental materials to explore the processing mechanism of sentence comprehension. The research design has two advantages. Firstly, QING structure is the one of the most representatively imperative sentences, and imperative sentence (S + V + O) is commonly used in Chinese daily communication (Zhang et al., 2013). The BCC corpus (/http://bcc.blcu.edu.cn/) reveals that the number of the Ba structural sentence is 15,251; the number of the Bei structural sentence is 7,100. However, for the Qing structure, there are 48,992 sentences [Qing: Qing (nr ns nt r n) v^*^n; Ba: (nr ns nt r n) Ba n^*^v 了; Bei: (nr ns nt r n) Bei n^*^v 了. nr represents personal name, ns represents place name, nt represents organization, r represents pronoun, n represents noun, and v represents verb]. Compared with the Ba and Bei structures, the Qing structure is used more frequently. Secondly, it is easier to construct syntactic vs. semantic violations in Qing structure than in Ba and Bei structures by only manipulating the verb between NP_1_ and NP_2_. For Ba or Bei structure, in order to construct syntactic vs. semantic violations, the researcher should control two conditions. One is that the NP following the preposition Ba or Bei requires to be definite. Another is the verb that should be limited to specific syntactic and semantic properties.

By manipulating the verb between NP_1_ and NP_2_ in In QING structure, we constructed four conditions, correct sentences (CORRECT) (e.g., 请你打扫这个房间。 Chinese Pinyin: Qing/ni/da/sao/zhe/ge/fang/jian. English translation: Please clean up this room), semantically violated sentences (SEMANTIC) by replacing the correct verb with a semantically unrelated but syntactically correct verb (e.g., 请你阅读这个房间。 Chinese Pinyin: Qing/ni/yue/du/zhe/ge/fang/jian. English translation: Please read this room), syntactically violated sentences (SYNTACTIC) by replacing the correct verb with a syntactically violated but semantic related word (e.g., 请你干净这个房间。 Chinese Pinyin: Qing/ni/gan/jing/zhe/ge/fang/jian. English translation: Please neat this room), and combined violated sentences (COMBINED) by replacing the correct verb with a both semantically and syntactically violated word (e.g., 请你幽默这个房间。 Chinese Pinyin: Qing/ni/you/mo/zhe/ge/fang/jian. English translation: Please humor this room), respectively.

In the initial phase (100–300 ms), according to the syntax-first model, the SYNTACTIC and COMBINED conditions would elicit an early anterior negativity (e.g., LAN) effect compared with the CORRECT condition, and this syntactic effect (e.g., SYSNTACTIC vs. CORRECT) would be earlier than the semantic effect (e.g., SEMANTIC vs. CORRECT). If these two aspects are independent and proceed in parallel, both syntactic and semantic violations would elicit an early effect, and there would be no interaction between these early syntactic and semantic effects. According to the interactive model, both the syntactic and semantic violations possibly elicit an early effect, and these syntactic and semantic processes are possible to interact with each other at the very early time window. Moreover, the relative beginning timing of the syntactic or semantic processing depends on which information comes first; that is, under specific circumstances, the semantic effect is likely to occur earlier than the syntactic effect. In the intermediate phase (300–500 ms), if Chinese sentence comprehension is similar to the syntax-first model, the N400 evoked by semantic violation in COMBINED would be suppressed. If not, we would see N400 in COMBINED. In the final phase (500–700 ms), if SYNTACTIC and COMBINED evoke P600, the processing of QING structure might be similar to Bei structure. If SYNTACTIC and COMBINED evoke negative waves in P600 time window, its processing might be similar to Ba structure. In general, if this processing in the three time-windows is different, we would expect that processing of Chinese QING sentence might be divided into different phases.

## Methods

### Subjects

There were 24 Tsinghua University students recruited to participate in the experiment (mean age = 22.126 years, range = 18–26 years, 12 males). All subjects have normal corrected vision (wearing glasses) and no physical and mental illness. They were all tested to be right–handed by the Edinburgh handedness test. None of them participate in the stimulus norming. All had signed the informed consent forms before starting the experiment. All the procedures of the experiment abided by the Declaration of Helsinki.

### Materials and Design

In the present study, the Chinese imperative QING sentences with the structure of QING + NP_1_ + V + NP_2_ were used as experimental stimuli. This sentence pattern represents a type of widely used imperative sentences in Chinese, in which the verb QING expressing the request precedes NP1. The arrangement of the target words in sentences was in reference to German and French original pieces of research (Friederici et al., 1999; Isel et al., 2007). All stimuli were consisted of 180 imperative sentences. The 180 experimental sentences can be divided into four different conditions: CORRECT, SEMANTIC, SYNTACTIC, and COMBINED, as shown in Table 1. All these 180 items were only appeared once. Each sentence consisted of eight Chinese characters. All the stimuli were designed to be easily understood in order to ensure that subjects could rapidly comprehend all sentences presented on the computer screen. Two stimulus normings were carried out to control the comprehensibility for four conditions and the semantic coherence between SEMANTIC and SYNTACTIC. In stimulus norming 1, a comprehension test was conducted to verify the degree of semantic violation in four conditions. Twenty-three subjects were recruited to judge the comprehensibility of the presented sentences. Score 1 indicated the sentence is completely incomprehensible, and score 5 indicated the opposite. (Bayes) two-way ANOVA test revealed a significant effect of semantic consistency [*F*_(1,176)_ = 1026.4, *p* < 0.001, BF_10_ = 7.9 × 10^51^], syntactic consistency [*F*_(1,176)_ = 185.9, *p* < 0.001, BF_10_ = 4.6 × 10^12^], and an interaction between semantic consistency and syntactic consistency [*F*_(1,176)_ = 99.0, *p* < 0.001, BF_incl_ = 2.9 × 10^15^). And a *post-hoc* test of Bayes one-way ANOVA, which used sentence type as a fixed factor, revealed that, except for SEMANTIC vs. COMBINED, there are significant differences between each pairwise comparison (CORRECT vs. SYNTACTIC: *p* < 0.001, BF_10,U_ = 3.1 × 10^49^; CORRECT vs. SEMANTIC: *p* < 0.001, BF_10,U_ = 1.4 × 10^63^; CORRECT vs. COMBINED: *p* < 0.001, BF_10,U_ = 3.6 × 10^89^; SEMANTIC vs. SYNTACTIC: *p* < 0.001, BF_10,U_ = 3.3 × 10^7^; SYNTACTIC vs. COMBINED: *p* < 0.001, BF_10,U_ = 1.2 × 10^14^). The average comprehensive values (standard error) of the four conditions were as follows: CORRECT: 4.824 (0.048), SEMANTIC: 1.942 (0.134), SYNTACTIC: 2.943 (0.180), COMBINED: 1.701 (0.133). Critically, compared with SEMANTIC and COMBINED, the result indicated that SYNTACTIC remained roughly comprehensible. In stimulus norming 2, 20 subjects who did not evaluate the comprehensibility were presented with SYNTACTIC. They were asked to replace the target word, which caused grammatical errors with correctly and semantically related words that emerged in their minds. The consistent rate between given answers and the corresponding words in correct sentences was 72.3%. Furthermore, 13.2% given answers were semantically related (not consistent) to target words in CORRECT (e.g., 删除/delete—清空/clear; 贡献/contribute—捐赠/donate). About 14.5% given answers were semantically unrelated to target words in CORRECT (e.g., 设计/design—修改/revise; 模仿/imitate—纠正/rectify). The result indicated a strong semantic correlation between CORRECT and SYNTACTIC, guaranteeing the obvious difference among SEMANTIC, SYNTACTIC, and COMBINED.

**Table 1:** Stimuli of Chinese imperative QING sentences for the four conditions with English translations.

a. CORRECT	请你/打扫/这个房间
	Please / clean up / this room.
b. SEMANTIC	请你/阅读/这个房间
	Please/ read / this room.
c. SYNTACTIC	请你/干净/这个房间
	Please / neat / this room.
d. COMBINED	请你/幽默/这个房间
	Please / humor / this room.

### Experiment Process

Subjects were requested not to take foods or drinks containing stimulants such as tea and coffee the day before the experiment. They were invited to sit in a quiet chamber far away from high-frequency radiation sources and noises. All stimuli were presented on the computer screen with a distance of 80 cm to subjects. The font of stimuli was white, courtier new, and size 50 with gray background. They were informed to quickly determine whether the Chinese imperative QING sentence that appeared on the screen was correct or not. There were five blocks in the whole experiment. The first block was the practice block, in which experimental results were not analyzed in the result part. There were 10 Chinese imperative QING sentences in total, of which the correct and incorrect sentences accounted for half. The next four blocks were formal experiments. In each block, 45 Chinese imperative QING sentences were presented in pseudorandom order. There were 180 sentences in the formal experiment. The presentation of each sentence was broken down into the following steps. Firstly, the fixation occurred in the middle of the screen for 400 ms to remind subjects to pay attention. Secondly, the first Chinese word of the sentence appeared in the center of the screen for 400 ms. Thirdly, the first word disappeared and the participants were left with an empty screen lasting 100 ms. Fourthly, the second word was presented on the screen for 400 ms. Finally, the target word appeared on the screen. After all the words were presented, the subjects were required to judge the sentence accuracy by using the experimental JoyStick. After finishing this task, the next trial started with fixation on the screen again.

### ERP Recordings and Analysis

The present study used a 62 Ag/AgCl electrodes elastic cap (Easycap; international 10–20 electrode placement system, Brain Products GmbH, Gilching, Germany) for electroencephalogram (EEG) data collection. FCz and AFz electrodes were regarded as reference and ground electrode, respectively. Besides, the vertical electrooculogram (VEOG) and the horizontal electrooculogram (HEOG) were respectively recorded through two ocular electrodes in the prescribed position. Resistances of electrodes were reduced under 20 KΩ. The BrainAmpDC amplifier system (Brain Products GmbH) with a bandpass of 0.01–100 Hz was configurated to amplify all raw EEG data. Then, all amplified data were aggregated into Brain Vision Recorder (Brain Products, Munich, Germany) software for further analysis (Zhang et al., 2020). All EEG data were bandpass filtered offline from 0.05 to 30 Hz. Any eye blinks or excessive movement (mean voltage exceeding ± 100 μV) was excluded. The observation window (200 ms pretarget baseline) was from the −200 ms before to 800 ms after the onset of the target word. In sum, EEG data of 24 subjects were computed.

Three time-windows were chosen according to previous studies (Friederici, 2002; Ye et al., 2006; Brouwer and Hoeks, 2013; Yang et al., 2015; Delogu et al., 2019; Zeng et al., 2020) and visual inspection for analysis: 100–300 ms time window for possible ELAN, 300–500 ms time window for possible N400 effect or LAN, and 500–700-ms time window for possible P600 effect, respectively. On the basis of the wide distribution of LAN, N400, and P600 in topographic maps, these ERPs components were analyzed for lateral electrodes, referring to previous sentence research. In each time window, repeated measures analysis of variance (ANOVA) was performed on mean amplitudes with four within-subject factors: semantic consistency (SEM+, SEM–), syntactic category (SYN+, SYN–), hemisphere (left, midline, and right), and region (anterior, central, and posterior). Laterality and anteriority were crossed to form nine regions of interest (ROI): left anterior (FP1, AF7, AF3, F1, F3, F5, F7, FC1, FC3, FC5, and FT7) (LA); midline anterior (FPz, Fz, and FCz) (MA); right anterior (FP2, AF4, AF8, F2, F4, F6, F8, FC2, FC4, FC6, and FT8) (RA); left central (C1, C3, C5, and T7) (LC); midline central (Cz and CPz) (MC); right central (C2, C4, C6, and T8) (RC); left posterior (CP1, CP3, CP5, TP7, P1, P3, P5, P7, PO3, PO7, and O1) (LP); midline posterior (Pz, POz, and Oz) (MP); and right posterior (CP2, CP4, CP6, TP8, P2, P4, P6, P8, PO4, PO8, and O2) (RP). Mean ERP amplitudes were averaged over the electrodes in each ROI (Yang et al., 2015). *Post-hoc* simple effect comparisons were conducted for critically significant interaction effects. However, since the frequentist approach provides only the evidence to reject the null hypothesis but not the alternative, Bayes factor was further adopted by performing Bayes repeated measure analysis. We calculated Bayes factor using the default priors (r scale fixed effects 0.5, r scale random effects 1, and r scale covariates 0.354) implemented in JASP (https://jasp-stats.org/). Frequentist *p* value was provided simultaneously to present significance, and Bayes factor was presented without threshold interpretation.

## Results

### Behavioral Data

**Figure 1:**
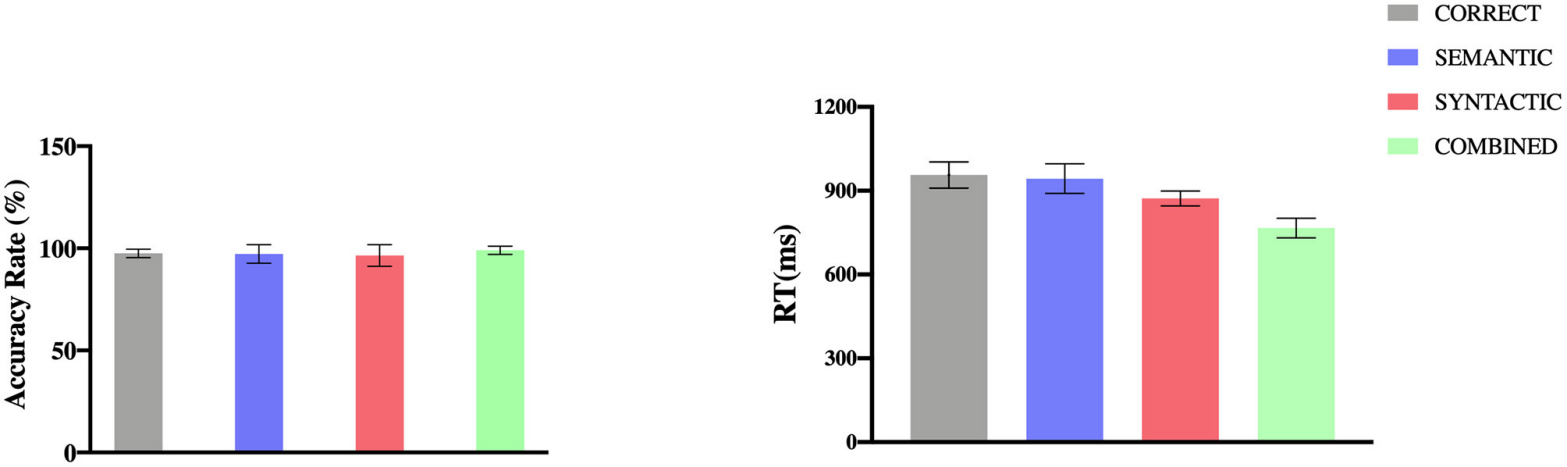
Accuracy rates and mean RT for the four conditions (error bars indicate standard error).

The entire response accuracy rate (ACC) was 97.50% across all four conditions: 97.50% for CORRECT (*SD* = 0.02); 97.22% for SEMANTIC (*SD* = 0.05); 96.39% for SYNTACTIC (*SD* = 0.05); 99.03% for COMBINED (*SD* = 0.02). A (Bayes) repeated-measures ANOVA with conditions showed no significant effect [*F*_(3,69)_ = 1.87, *p* = 0.143, BF_10_ = 0.58]. The overall mean reaction time (RT) was 908.22 ms across all four conditions: 956.21 ms for CORRECT (*SD* = 36.78); 943.21 ms for SEMANTIC (*SD* = 52.98); 872.31 ms for SYNTACTIC (*SD* = 27.03) and 766.43 ms for COMBINED (*SD* = 34.97). However, according to previous pieces of researches (Friederici et al., 2004; Zhang et al., 2010) which did not measure RTs, we consider that the RT was not informative in the current study because it was recorded long after the appearance of sentence stimuli. Both ACC and mean RT were shown in Figure 1. In general, the whole behavioral results showed that all subjects were attentive to each trial in the experiment.

### Electrophysiological Data

**Figure 2:**
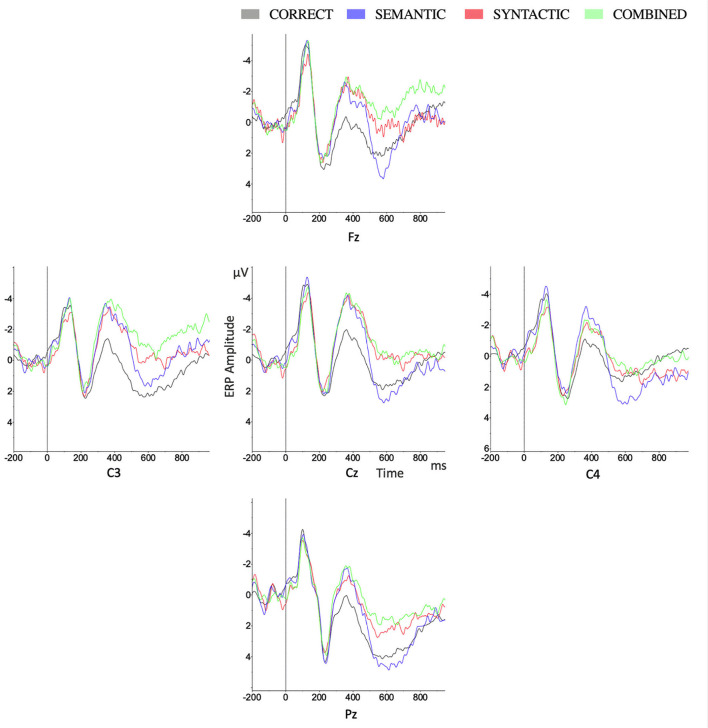
Grand average ERPs for four conditions on lateral (C3, C4) and midline (Fz, Cz, Pz) electrodes.

**Figure 3:**
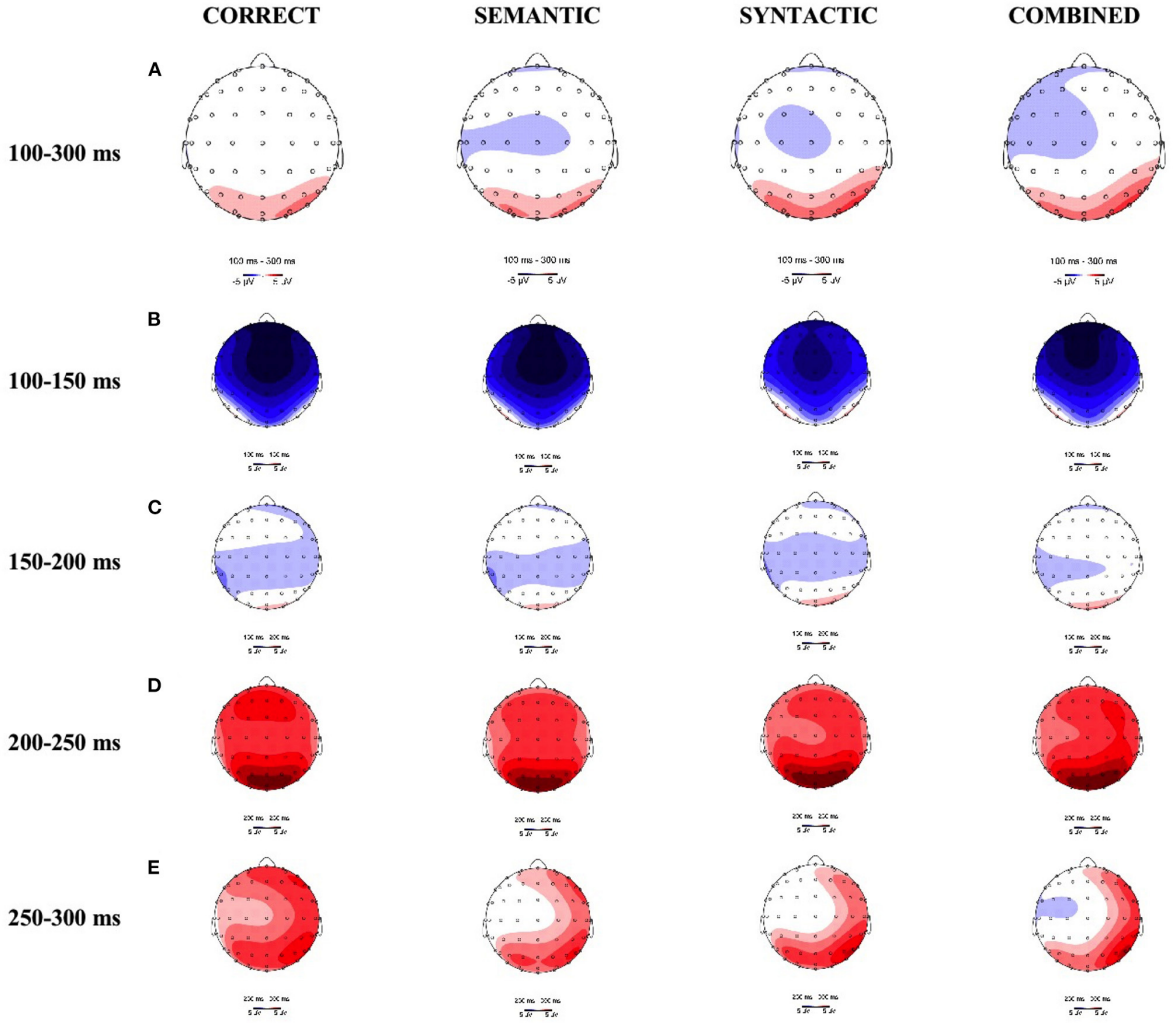
Topographic distributions of the ERP differences at **(A)** the 100–300 ms, including **(B)** the 100–150 ms, **(C)** the 150–200 ms, **(D)** the 200–250 ms, and **(E)** the 250–300 ms windows, respectively.

**Figure 4:**
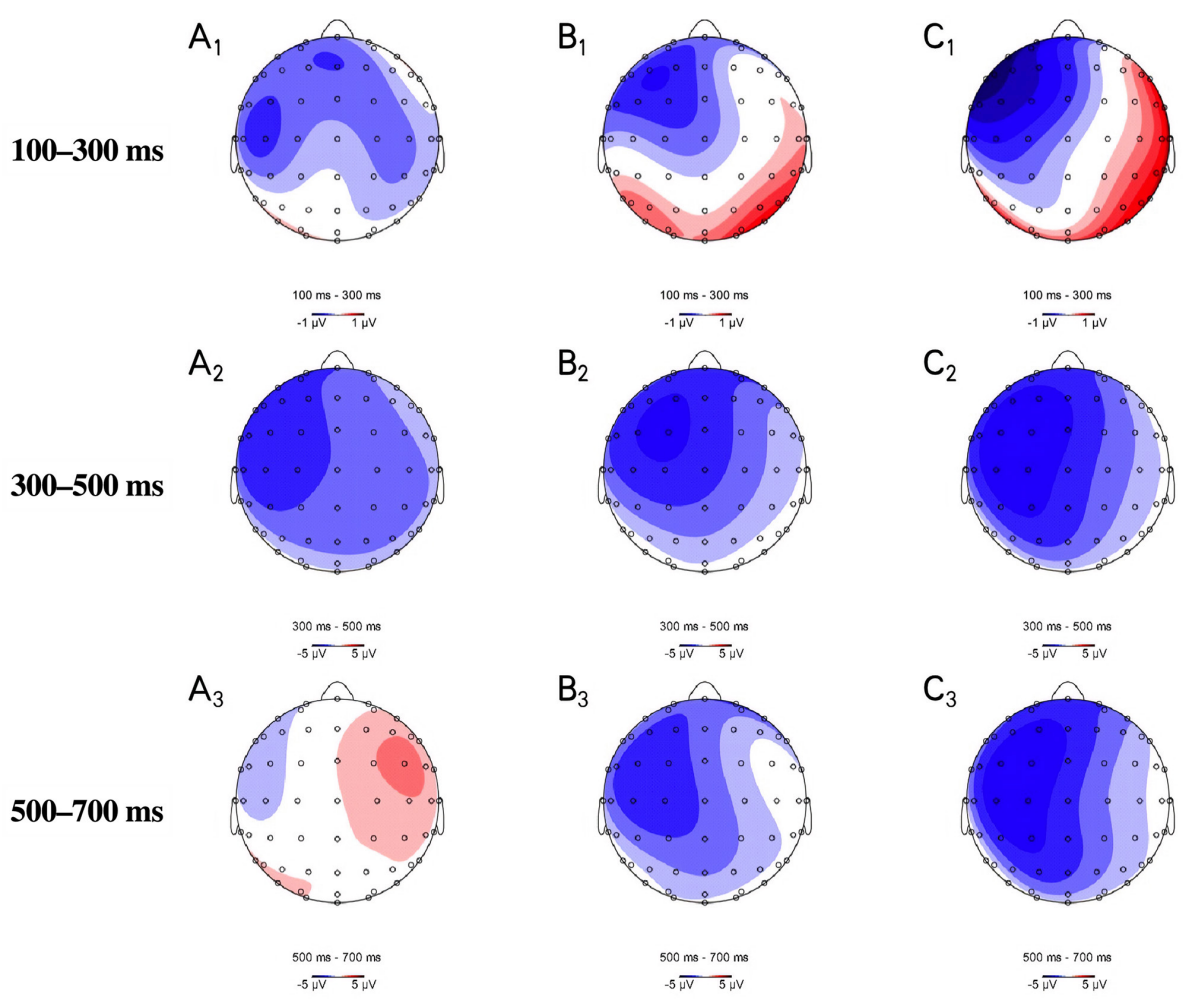
Topographic distributions of the ERP differences at the 100−300, 300–500 and 500–800 ms windows, respectively. **(A)** SEMANTIC, **(B)** SYNTACTIC, and **(C)** COMBINED. The three violated conditions were all compared with the CORRECT condition.

As shown in Figures 2, 3, 4 in the time window of 100–300 ms, CORRECT, SYNTACTIC, SEMANTIC, and COMBINED showed marginally different amplitudes. In the time window of 300–500 ms, different from the CORRECT, violated sentences, including SYNTACTIC, SEMANTIC, and COMBINED, evoked obvious negative waves (N400 effect), respectively. These negative effects for both SEMANTIC and SYNTACTIC were largely on the left hemisphere shown in Figure 4 with similar distribution over the scalp. For COMBINED, the N400 effect was distributed almost over the whole scalp. In the 500–700 ms time window, only the CORRECT stimulated a small positive wave in the right anterior region. Surprisingly, there was not any significant positive effect in either SYNTACTIC or COMBINED. Statistical analyses were performed to confirm these observations.

#### The 100–300 ms Time Window

A (Bayes) repeated measure ANOVA revealed an interaction between SYNTACTIC consistency and region [*F*_(2,46)_ = 4.4, *p* = 0.02, BF_incl_ = 1.89, see Figure 3A], and an interaction between SYNTACTIC consistency and SEMANTIC consistency [*F*_(1,23)_ = 8.4, *p* = 0.008, BF_incl_ = 2.49 × 10^7^] during 100–300 ms time window. In the case of syntactic correction, SEMANTIC evoked a marginally more negative effect than CORRECT [*F*_(1,23)_ = 32, *p* = 0.087, BF_10_ = 1.9 × 10^5^]. In the case of syntactic violation, the difference between SYNTACTIC and COMBINED was not significant [*F*_(1,23)_ = 2.2, *p* = 0.149, BF_10_ = 2.9 × 10^2^]. In the case of semantic correction, the difference between CORRECT and SYNTACTIC was not significant [*F*_(1,23)_ = 0.7, *p* = 0.410, BF_10_ = 0.5]. In the case of semantic violation, SEMANTIC evoked a significantly more negative effect than COMBINED [*F*_(1,23)_ = 9.8, *p* = 0.005, BF_10_ = 3.6 × 10^12^].

To further examine whether there was any significant difference between SEMANTIC consistency and SYNTACTIC consistency between 100–300 ms, we subdivided the time window by 50-ms time intervals to get four sub-windows: 100–150, 150–200, 200–250, and 250–300 ms for more accurate statistics. Within each sub-window, (Bayes) repeated measure ANOVA showed that SYNTACTIC consistency constantly interacted with region [100–150 ms: *F*_(2,46)_ = 5.2, *p* = 0.009, BF_incl_ = 3.1, see Figure 3B; 150–200 ms: *F*_(2,46)_ = 4.6, *p* = 0.015, BF_incl_ =1.5, see Figure 3C; 200–250 ms: *F*_(2,46)_ = 4.4, *p* = 0.017, BF_incl_ =1.6, see Figure 3D; 250–300 ms: *F*_(2,46)_ = 3.3, *p* = 0.044, BF_incl_ = 0.8, see Figure 3E], and with SEMANTIC consistency [100–150 ms: *F*_(1,23)_ = 7.3, *p* = 0.013, BF_incl_ = 2.0 × 10^6^; 150–200 ms: *F*_(1,23)_ = 9.2, *p* = 0.006, BF_incl_ = 5.1 × 10^7^; 200–250 ms: *F*_(1,23)_ = 7.7, *p* = 0.011, BF_incl_ = 1.3 × 10^7^; 250–300 ms: *F*_(1,23)_ = 8.6, *p* = 0.008, BF_incl_ = 1.6 × 10^8^]. In the case of syntactic correction, SEMANTIC always elicited marginally larger negative effect than CORRECT [100–150 ms: *F*_(1,23)_ = 2.5, *p* = 0.125, BF_10_ = 1.9 × 10^4^; 150–200 ms: *F*_(1,23)_ = 3.3, *p* = 0.081, BF_10_ = 5.0 × 10^5^; 200–250 ms: *F*_(1,23)_ = 3.3, *p* = 0.084, BF_10_ = 1.6 × 10^5^; 250–300 ms: *F*_(1,23)_ = 3.6, *p* = 0.072, BF_10_ = 3.7 × 10^5^]. In the case of syntactic violation, the differences between SYNTACTIC and COMBINED were not significant [100–150 ms: *F*_(1,23)_ = 2., *p* = 0.175, BF_10_ = 1.2 × 10^2^; 150–200 ms: *F*_(1,23)_ =2.2, *p* = 0.154, BF_10_ = 249.; 200–250 ms: *F*_(1,23)_ = 2.1, *p* = 0.158, BF_10_ = 203.2; 250–300 ms: *F*_(1,23)_ = 2.6, *p* = 0.121, BF_10_ = 9.7 × 10^2^]. In the case of semantic correction, the difference between CORRECT and SYNTACTIC did not reach significance [100–150 ms: *F*_(1,23)_ = 0.3, *p* = 0.579, BF_10_ = 0.2; 150–200 ms: *F*_(1,23)_ = 0.6, *p* = 0.440, BF_10_ = 0.4; 200–250 ms: *F*_(1,23)_ = 0.8, *p* = 0.375, BF_10_ = 0.6; 250–300 ms: *F*_(1,23)_ = 1.1, *p* = 0.302, BF_10_ = 1.193]. In the case of semantic violation, SEMANTIC consistently evoked more negative effect than COMBINED [100–150 ms: *F*_(1,23)_ = 9.7, *p* = 0.005, BF_10_ = 1.6 × 10^12^; 150–200 ms: *F*_(1,23)_ = 10.8, *p* = 0.003, BF_10_ = 5.0 × 10^13^; 200–250 ms: *F*_(1,23)_ = 8.6, *p* = 0.007, BF_10_ = 1.6 × 10^11^; 250–300 ms: *F*_(1,23)_ = 9.4, *p* = 0.006, BF_10_ = 2.4 × 10^12^].

#### The 300–500 ms Time Window

A (Bayes) repeated measure ANOVA revealed significant main effects of region [*F*_(2,46)_ = 12.1, *p* < 0.001, BF_10_ = 5.2 × 10^12^], hemisphere [*F*_(2,46)_ = 4.6, *p* = 0.016, BF_10_ = 1.0 × 10^1^], and a marginally significant interaction between SYNTACTIC consistency and SEMANTIC consistency [*F*_(1,23)_ = 3.0, *p* = 0.096, BF_incl_ = 13.5]. When semantically correct, SYNTACTIC elicited a larger negative effect than CORRECT [*F*_(1,23)_ = 5.853, *p* = 0.024, BF_10_ = 4.7 × 10^6^]. In the case of semantic violation, the difference of amplitude between SEMANTIC and COMBINED was not significant [*F*_(1,23)_ = 0.012, *p* = 0.913, BF_10_ = 1.3]. When syntactically correct, SEMANTIC showed a larger negative effect than CORRECT [*F*_(1,23)_ = 8.5, *p* = 0.008, BF_10_ = 8.9 × 10^9^]. In the case of syntactic violation, the difference of amplitude between SYNTACTIC and COMBINED was not significant [*F*_(1,23)_ = 0.092, *p* = 0.765, BF_10_ = 0.13].

To further compare three violated sentence types, we subtracted the ERP amplitude of CORRECT sentences from each violated sentence and performed Bayes three-way repeated measure ANOVA (hemisphere × region × sentence type) on the remaining ERP component of three violated sentence types. Results revealed that there was no significant sentence type effect [*F*_(2,46)_ = 0.032, *p* = 0.969, BF_10_ = 0.023] within these three violated sentence types.

#### The 500–700 ms Time Window

A (Bayes) repeated-measures ANOVA revealed significant main effect of syntactic consistency[*F*_(1,23)_ = 7.24, *p* = 0.013, BF_10_ = 1.3 × 10^10^], region [*F*_(2,46)_ = 15.4, *p* < 0.001, BF_10_ = 9.4 × 10^8^], hemisphere [*F*_(2,46)_ = 3.4, *p* = 0.041, BF_10_ = 7.8 × 10^0^]. Compared with syntactically correct sentences, syntactically violated sentences evoked a more negative ERP component (*p* = 0.013, BF_10,U_ = 3.7 × 10^9^). Spatially, more positive amplitudes appeared on right hemisphere (right vs. midline: *p* = 0.845 BF_10,U_ = 0.079; right vs. left: *p* = 0.067, BF_10,U_ = 705.5) and posterior region (posterior vs. anterior: *p* < 0.001, BF_10,U_ = 1.3 × 10^14^; posterior vs. center: *p* < 0.001, BF_10,U_ = 2.9 × 10^17^). Meanwhile, frequentist analysis revealed interactions between hemisphere × region [*F*_(4,92)_ = 3.842, *p* = 0.006, BF_incl_ = 0.028] and hemisphere × region × semantic consistency [*F*_(4,92)_ = 4.631, *p* = 0.002, BF_incl_ = 0.017]. However, both interactions were not supported by the Bayes factor.

## Discussion

The present study adopted ERPs to investigate the processing mechanism of Chinese sentence processing by using Qing structures. Behavioral results indicated that most subjects completed each trial carefully and attentively. Main results were as follows: In the 100–300 ms time window, there existed an interaction between SEMANTIC consistency and the SYNTACTIC category. In the 300–500 ms time window, the interaction continued with similar negative waves evoked by three types of violated sentences. In the 500–700 ms time window, while there appeared obvious negative waves rather than P600 in SYNTACTIC or COMBINED, the main effect of SYNTACTIC consistency was also significant. Overall, the findings suggested that the comprehension of Chinese Qing structure might be subdivided into different phases, and its processing mechanism was similar to the interactive model. The processing differences between Chinese and Indo-European languages, as well as within Chinese, are discussed below.

### The 100–300 ms Time Window

The amplitude during 100–300 ms time window in our study seemed different from that of Indo-European languages. Previous studies using German as material evoked an obvious early negativity (similar to ELAN) in both SYNTACTIC and COMBINED, which included a syntactic violation (Friederici et al., 1999; Friederici, 2002). The ELAN was reported to restrain the amplitude of N400 in the following step evoked by semantic violations in COMBINED, which was regarded as evidence that supported the syntax-first model. Although we also found a negative wave in the same time window, there was not enough evidence to speculate that it was ELAN or LAN. In addition, we found a significant interaction between semantic and syntactic factors. What is more, we subdivided the time window into four parts for more accurate statistics and still found significant interactions in each time interval.

The difference between Chinese and Indo-European languages may be mainly due to the following three aspects: Firstly, Indo-European language words were equipped with complex grammatical inflections, which might mark a word category and a grammatical feature in a sentence. Such a syntactic category of words is likely to be distinguished by grammatical structures or morphological forms (e.g., act, action, actor, active, and actively). Based on these characteristics, syntactic processing might play a dominant part in sentence comprehension. In contrast, such properties that might be applied for real-time verb-tense retrieval and phrase grouping are mostly unavailable in Chinese (Li et al., 1993). Secondly, Indo-European languages are likely to encode thematic-role information in morphemes neighborly attached to referential noun phrases. However, the Chinese language lacks a mature morphosyntactic system, thus probably restraining the commitment to immediate constituent attachment in favor of a more conservative strategy that looks for more detailed information from sentences in each processing. What is more, as a paratactic language, Chinese syntactic structure building might depend mainly on the lexical-semantic and contextual meaning of words in a sentence. Therefore, semantic processing may have distinguishing values and even play different roles in different types of language.

The processing in the initial phase of the present study seems also different from that of Chinese Ba structure (Ye et al., 2006). While (Ye et al., 2006) found that syntactic processing and semantic processing for Ba structure were independent and parallel, our study showed that there was a continuous interaction between these two kinds of processing during 100–300 ms time window, which might be similar to the interactive model. Given that Chinese sentence processing may be inclined to be interactive or parallel in this time window, it can be speculated that different sentence structures may be processed differently in the initial phase.

Unexpectedly, we found obvious N1 (100–150 ms) component in three violated sentences, similar to Previous Ba structure (Zhang et al., 2010). Previous studies explained this phenomenon as follows: Since N1 was likely to reflect the attentional effort of individuals, the more comprehensible the context was, the more attention the individual paid. In the current study, since there was a significant interaction of comprehensibility between SEMANTIC consistency and a SYNTACTIC category, and also a significant interaction of N1 between SEMANTIC consistency and a SYNTACTIC category, we speculated that the N1 may be related to the comprehensibility. As N1 was reported to reflect the earlier and more underlying processing, we did not have enough evidence to convey that it reflected the processing of either semantics or syntax. This phenomenon is worth further exploring.

### The 300–500 ms Time Window

Unlike previous German research in which N400 was suppressed in the 300–500 ms time window for COMBINED, this violated type in our research evoked a significant negative amplitude. Such evidence might also convey that semantic processing was likely not impeded by syntactic violation. In addition, our results were similar to that of Ba structure (Ye et al., 2006),with three violated sentences evoked obvious negative waves. However, those negative waves caused by SYNTACTIC and COMBINED of Ba structures were regarded as LAN (Ye et al., 2006); we were unsure if these negative waves evoked by these two types of violation in the present study were LAN. Further spatial localization evidence is needed to explain this question in the future.

Results also revealed that there was no significant difference within these three violated sentences during 300–500 ms time window, although their comprehensibility was obviously different except for SEMANTIC vs. COMBINED. By comparison, in previous Ba (Ye et al., 2006, 2007; Zhang et al., 2010) and Bei structures (Yang et al., 2015), the differences of comprehensibility for each violation were consistent with their N400 amplitudes. Although previous studies have reported a close relationship between the magnitude of N400 and sentence comprehensibility, there was a limitation that we were not sure about what caused the result based on the existing evidence. Whether it might be also influenced by sentence structures is a matter for further study.

### The 500–700 ms Time Window

Neither SYNTACTIC nor COMBINED evoked P600 in the final phase. Instead, both of them elicited significant negative amplitudes. This result seemed similar to that of some Ba structures (Ye et al., 2006, 2007), however different from those of other Ba structures (Yu and Zhang, 2008; Zhang et al., 2010) and Bei structures (Yang et al., 2015; Zeng et al., 2020). Ye et al. (2006) gave explanations of the absence of P600 as follows: First, SYNTACTIC might be affected by possible overlap between the largely distributed later negativity and the posterior positivity. Second, COMBINED might be influenced by a feasible overlap between the wrap-up effect (Hagoort, 2003) and the posterior positivity. However, despite the exclusion of the wrap-up effect, P600 was still absent in this time window (Ye et al., 2007). We were not sure whether the absence of P600 in the present study was caused by similar reasons. Sentence processing seemed more complicated than previously expected during this time window. Different constructions may be processed differently, meanwhile sharing possible processing mechanisms. It is worth investigating how functionally important such a shared processing mechanism is in the further study.

## Conclusion

To conclude, the present study used Qing structure to provide new evidence for the processing mechanism of Chinese sentence patterns. Specifically, we found that the interactive model rather than the syntax-first model may apply to the processing of this specific structure of Chinese sentence. Meanwhile, the interaction between SEMANTIC consistency and the SYNTACTIC category appeared in the initial phase, which appeared different from Ba structures (Ye et al., 2006). Compared with the results of previous studies, we speculated that the processing of Chinese might be complicated. It is likely that different sentence structures have different processing mechanisms. How such differences occur is a topic worthy of research attention in the future.

## Data Availability Statement

The original contributions presented in the study are included in the article/supplementary material, further inquiries can be directed to the corresponding author.

## Ethics Statement

The studies involving human participants were reviewed and approved by the Ethics Committee of the School of Psychology at Tsinghua University. The patients/participants provided their written informed consent to participate in this study.

## Author Contributions

SY did literature research, conceived, designed the experiments, performed the experiments, finished data collection, and wrote the paper. YC performed the pretest, performed some experiments, and analyzed the data. WX reviewed the manuscript, gave constructive suggestions, and proofread the whole manuscript. MJ provided laboratory, equipment and software, and reviewed the manuscript. All authors contributed to the article and approved the submitted version.

## Funding

This work was supported by National Natural Science Key Foundation of China, Research on Cognitive Mechanism and Computational Model of Language Understanding (62036001) and National Social Science Major Foundation of China, Research on Advanced Cognition at the Level of Language, Thought, and Culture (15ZDB017).

## Conflict of Interest

The authors declare that the research was conducted in the absence of any commercial or financial relationships that could be construed as a potential conflict of interest.

## Publisher's Note

All claims expressed in this article are solely those of the authors and do not necessarily represent those of their affiliated organizations, or those of the publisher, the editors and the reviewers. Any product that may be evaluated in this article, or claim that may be made by its manufacturer, is not guaranteed or endorsed by the publisher.
